# Angiotensin II system in the nucleus tractus solitarii contributes to autonomic dysreflexia in rats with spinal cord injury

**DOI:** 10.1371/journal.pone.0181495

**Published:** 2017-07-24

**Authors:** Kai Wang, Shaoxia Duan, Xueping Wen, Weizhong Wang, Shangping Fang, Dunyi Qi, Xiang Huan, Liwei Wang, Zhenzhou He

**Affiliations:** 1 Department of Anesthesiology, Central Hospital of Xuzhou, Jiangsu, China; 2 Department of Anesthesiology and ICU, South Campus, Ren Ji Hospital, School of Medicine, Shanghai Jiao Tong University, Shanghai, China; 3 Department of Orthopedics, Ningxiang People’s Hospital of Hunan Province, Ningxiang, Hunan, China; 4 Department of Physiology, Second Military Medical University, Shanghai, China; 5 Department of Anesthesiology, Changzheng Hospital, Second Military Medical University, Shanghai, China; 6 Department of Anesthesiology, Affiliated Hospital of Xuzhou Medical University, Jiangsu, China; Max Delbruck Centrum fur Molekulare Medizin Berlin Buch, GERMANY

## Abstract

**Background:**

Autonomic dysreflexia (AD) is a potentially life-threating complication after spinal cord injury (SCI), characterized by episodic hypertension induced by colon or bladder distension. The objective of this study was to determine the role of impaired baroreflex regulation by the nucleus tractus solitarii(NTS) in the occurrence of AD in a rat model.

**Methods:**

T4 spinal cord transection animal model was used in this study, which included 40 Male rats Colorectal distension (CD) was performed to assess AD and compare the changes of BP, HR, and BRS, six weeks after operation. After that, SCI rats with successfully induced AD were selected. Losartan was microinjected into NTS in SCI rats, then 10, 30, 60 minutes later, CD was performed to calculate the changes of BP, HR, and BRS in order to explicit whether Ang II system was involved in the AD occurrence. Ang II was then Intra-cerebroventricular infused in sham operation rats with CD to mimic the activation of Ang II system in AD. Finally, the level of Ang II in NTS and colocalization of AT1R and NMDA receptor within the NTS neurons were also detected in SCI rats.

**Results:**

Compared with sham operation, SCI significantly aggravated the elevation of blood pressure (BP) and impaired baroreflex sensitivity (BRS) induced by colorectal distension; both of which were significantly improved by microinjection of the angiotensin receptor type I (AT_1_R) antagonist losartan into the NTS. Level of angiotensin II (Ang II) in the NTS was significantly increased in the SCI rats than sham. Intracerebroventricular infusion of Ang II also mimicked changes in BP and BRS induced by colorectal distension. Blockade of baroreflex by sinoaortic denervation prevented beneficial effect of losartan on AD.

**Conclusion:**

We concluded that the activation of Ang II system in NTS may impair blood pressure baroreflex, and contribute to AD after SCI.

## Introduction

Autonomic dysreflexia (AD) commonly occurs in patients with spinal cord injury (SCI) at or above the level of the sixth thoracic segment(T6)[1]. An episode of AD is characterized by huge elevation of blood pressure (BP) with bradycardia or sometimes tachycardia. It is accepted that an increase greater than 20–30 mmHg in systolic BP could be considered as AD[2]. In a subgroup of SCI patients, especially the ones with cervical and high thoracic SCI, a dysreflexic episode could be missed, for it only appears in normal or slightly elevated ranges, as the resting BP in these patients is often lower than that in normal individuals[3]. Often, the episodic hypertension is triggered by urinary bladder or colon irritation[2,4].

Although AD usually behaves as a mild discomfort, it can also cause intracranial hemorrhage, coronary artery constriction, retinal detachments, pulmonary edema, etc.[5,6], and becomes a devastating event. It is observed that the incidence and severity of AD is affected by several factors, such as the level of injury, the completeness of injury, and the time after injury[2]. It is reported that AD occurs in up to 70% patients with quadriplegia and high paraplegia, in the stage of chronic SCI[7,8], and is the primary cause of morbidity and mortality[9,10].

Several hypotheses have been proposed for the development of AD. Plastic changes within the spinal cord and peripheral autonomic circuits are thought to be the main reason for autonomic instability which leads to elevated BP[11]. The loss of supraspinal input contribute to the situation[12]. Altered sensitivity of peripheral alpha-adrenergic receptors is also one possible explanation[13]. All these proposed mechanisms focus on the changes of the peripheral nervous system. Despite there are a few articles reported that propriospinal changes as well as interneuronal sensitivity alterations following spinal cord injury[14,15], interruption of tonically active medullo-spinal pathways[16], and sprout of the primary afferent fibers[17]are probably involved, but what role does the central nervous system play in the progression of AD remains partly undetermined.

It is well known that the BP baroreflex, a negative feedback loop would rapidly respond to an elevated BP in a healthy body. A reasonable inference that impaired baroreflex sensitivity (BRS)may function in patients with SCI has been proven[18]. Nucleus tractus solitarii (NTS), a relay station of baroreflex transmission, plays a key role in tonic and reflex control of cardiovascular activity. It is found that abnormalities in the NTS inhibit BRS and lead to severe hypertension[19,20], and an increase of angiotensin II (Ang II) in the NTS is relevant[21,22]. It is reported that the activation of central angiotensin II system in NTS participants in this hypertension [23,24]. Microinjection of angiotensin II into NTS has been found to depress the baroreflex function, whereas microinjection of the antagonist of angiotensin II primary receptor, angiotensin II receptor 1 (AT1R), could facilitate the BRS[25].

In present study, the principle goal was to clarify whether the blunted BRS function involved in the hypertension induced by AD, and the relationship between AD and angiotensin II system in NTS. We used nitroprusside sodium and phenylephrine injection to measure the values of BRS in SCI baseline and during AD which was triggered by colon distension. We also investigated the effect of bilateral microinjection of the AT1R blocker-losartan into the NTS on the elevated BP of AD. Furthermore, the experiments were duplicated and followed by sinoaortic denervation in SCI rats and intracerebroventricular administration of angiotensin II in intact rats to test and verify our hypothesis.

## Materials and methods

### Animals

All studies were conformed to the Guide for the Care and Use of Laboratory Animals published by the US National Institutes of Health (NIH Publication No. 85–23, revised 1996) and approved by the Institutional Care and Use Committee of the Second Military Medical University. A total of 40 Male Sprague-Dawley rats (Sino-British SIPPR/BK Laboratory Animal Ltd, Shanghai, China) weighing between 300 and 350 g were enrolled in the study and housed in a light and temperature-controlled cage with food and water available *ad libitum*.

### Model preparation

T4 spinal cord transection animal model was used in this study in accordance with other researches[26]. Rats were anesthetized with 3% isoflurane. After median incision of the back and dissection of the fascia and muscles in layers, a T3 vertebra laminectomy was performed to expose the T4 spinal cord. In the SCI group, both the dura mater and spinal cord were completely transected with micro-scissors. The completeness of transection was verified by visual inspection of the lesion site. Absorbable gelatin sponge was inserted into the gap to reduce bleeding, and was laid over the vertebral canal to protect the spinal cord. In the control group, the T4 spinal cord was exposed only without transection. All the operative procedures were performed with aseptic surgical techniques. Penicillin was used for three days after the operation. During the recovery period (about one week), all rats were taken care of three times a day. Physical manipulations were performed to prevent pressure sores and gentle manual compressions were used to avoid bladder filling until the rats could accomplish automatic micturition. All rats were allowed to recover for six weeks.

### Surgery and experimental protocol six weeks after SCI

Six weeks after T4 transection, 30SCI and 20sham operation rats were anesthetized with intraperitoneal α-chloralose (40 mg/kg) and urethane (800 mg/kg). The trachea was cannulated and artificial ventilated with mixed 100% oxygen and room air for assistance. The left femoral artery was cannulated using polyethylene tubing PE10 (Smiths medical Company, UK) to record BP and heart rate (HR) by PowerLab/8PS (AD Instruments, Australia). Both the left and right femoral vein were cannulated using polyethylene tubing PE50 for fluid and drug infusion. Rats were placed in a stereotaxic frame (Narishige, Japan) and the dorsal surface of the medulla oblongata was exposed by removing part of the occipital bone and dura from incising of the atlantooccipital membrane. Temperature of the rats was maintained at about 37°C with a temperature controller (World Precision Instruments, USA). Colorectal distension (CD) was performed in SCI and sham operation rats to assess AD and compare the changes of BP, HR, and BRS in the two groups. After that, SCI rats with successfully induced AD were selected for the following experiments. Losartan was microinjected into NTS in SCI rats, then 10, 30, 60 minutes later, CD was performed to calculate the changes of BP, HR, and BRS in order to explicit whether Ang II system was involved in the AD occurrence. Ang II was then Intra-cerebroventricular infused in sham operation rats with CD to mimic the activation of Ang II system in AD. For the further explanation of the function of baroreflex in AD occurrence, Sinoaortic denervation was used in SCI rats. Finally, the level of Ang II in NTS and colocalization of AT1R and NMDA receptor within the NTS neurons were also detected in SCI rats.

### Assessing AD triggered by colorectal distension

AD in rats was assessed six weeks after SCI or sham operation[4]. After anesthesia, CD was performed using a latex balloon-tipped Swan-Ganz catheter (Edwards LifeSciences, USA). The catheter was inserted into the rectum for 2 cm, fixed to the tail with tape, and left in place for about 10 min until BP became stable. The volume of 2 ml air was syringed to inflate the balloon catheter for about 1 min to mimic the noxious colorectal distension[27]. This volume of air generated a pressure of about 35mmHg, measured through a sidearm of the catheter. The colorectal distension was performed twice with an interval of 10min.The BP values before and after colorectal distension were recorded twice and averaged, respectively. An elevation greater than20% from baseline was considered as AD status[2],the rats failed to induce AD were excluded from the following experiments.

### NTS microinjections

The effect of losartan (an AT1R antagonist) on changes in BP and BRS after microinjection into the NTS was explored with SCI rats in which AD can be observed. NTS Microinjection (volume of 50 nl) was achieved with three-barrel micropipettes (20–30 μm tips) using a pneumatic pressure injector (World Precision Instruments, USA) 10 minutes after anesthesia. The position of NTS was located as 0.4–0.5 mm rostral, 0.5–0.6 mm lateral, and 0.4–0.5 mm deep to calamus scriptorius, and functionally identified by a rapid depressor response (>25 mmHg) to 1 nmol L-glutamate injection[28]. The interval of 60 s existed between bilateral injections. After identification of NTS with 10 minutes washout, 1.6 nmol losartan was injected for follow up experiments. L-glutamate and losartan were dissolved in artificial cerebrospinal fluid (aCSF). The vehicle injection had performed as a control to prevent the interference. The injection sites were confirmed by microinjection of 50 nl 2% pontamine sky blue for analysis at the end of each experiment.

### Measurement of baroreflex sensitivity

The most common method for quantitatively assessing BRS is to alter BP pharmacologically, based on the Oxford and Modified Oxford techniques[29]. In our study, nitroprusside sodium (100 μg/kg) was used to decrease BP (40–50 mmHg), and then increased it (140–150 mmHg) with phenylephrine (80 μg/kg)[28]. The BRS was calculated as the ratio of changes in HR (beat/min) and changes in MAP (mmHg) (ΔHR / ΔMAP).

### Intra-cerebroventricular infusion

This experiment was performed to verify the effect of central Ang II on MAP elevation induced by the colorectal distension. After anesthesia, the atlantooccipital membrane was exposed and the fourth ventricle was punctured into by a stainless-steel cannula, verified by effusion of the cerebrospinal fluid. The cannula was connected to a 0.5 ml syringe via a 30 cm flexible tube. Ang II was infused at a rate of 300 μl/h for one hour and the concentration was 150 pmol/100 μl. The MAP and HR in response to colorectal distension were observed 30 min and 60 min after central infusion of Ang II.

### Sinoaortic denervation (SAD)

SAD eliminates the baroreflex by removing afferent inputs from arterial baroreceptors[30]. After anesthesia, atropine (0.5 mg/kg) was used to prevent salivary secretion in rats with AD observed. Followed by a midline incision, bilateral superior laryngeal nerves were sectioned. Connective tissue of carotid bifurcation regions was stripped and the area was painted with 10% phenol in ethanol. After the SAD surgery, the baroreceptor denervation was accepted as the decrease in HR was no more than 6 bpm in response to phenylephrine[31].

### Measurement of Ang II

Rats were euthanized with an overdose of pentobarbital sodium (200 mg/kg) and removed the brain, which were frozen rapidly with liquid nitrogen. NTS tissues were punched according to rat atlas in cycstat, lysed with lysate, and sonicated the mixture. The supernatants were extracted for detecting the level of Ang II after centrifugation. Level of Ang II was measured by the Elisa kits (no.F15050, Shanghai Westang Biotech CO., LTD) according to the manufacturer’s instructions. In brief, protein samples were added to coated wells for 40 minutes at 37°C, washed wells 3 times, and added biotinylated antibody for 20 minutes at 37°C, wells washed 3 times, reacted with horseradish peroxidase conjugated secondary antibody, and added with substrates (TMB Solution). The concentration of Ang II were determined the absorbance at 450 nm with an automated micro plate reader, and were calculated according to the standard curve. The level of Ang II in the NTS was expressed as the ratio of the concentration of Ang II to the concentration of total protein in protein samples.

### Immunohistochemisty

In order to detect the colocalization of AT1R and NMDA receptor within the NTS neurons, a double staining immunohistochemistry was performed. The rats were euthanized by an overdose of pentobarbital sodium (200 mg/kg) and perfused through the aorta with 0.9% NaCl solution and 4% paraformaldehyde in 0.1 mol/L phosphate buffer. The brains were removed and post-fixed in 4% paraformaldehyde in 0.1 mol/L phosphate buffer, overnight. The brain blocks were then transferred to 20% sucrose in phosphate-buffered saline (PBS) and kept in the solution until they sank to the bottom. Then, the brain blocks were rapidly frozen. Sections of 20 μm thickness were cut in a cryostat and floated in PBS. The sections were pre-incubated in antibody dilution solution (5% Bovine Albumin V, 0.2% Triton X-100 and 0.05% sodium azide in PBS) for 30 min at room temperature(RT), followed by incubation with the 1st primary antibody, NMDAR1 antibody (Abcam) overnight at 4°C. Subsequently, the sections were incubated with FITC-conjugated IgG. The sections were then incubated with the 2nd primary antibodies of AT1R (SIGMA-ALORICH, Anti-AGTR1/AT1) overnight at 4°C. Subsequently the sections were incubated with TRITC-conjugated IgG. All the incubations and reactions were separated by 5 min washes (3 times) in PBS wash buffer. Finally, the sections were then mounted on slides and embedded. Images were taken with the Olympus digital camera DP72 (Olympus, Japan) attached to an Olympus microscope (IX71, Olympus, Japan).

### Statistical analysis

All data were presented as mean±SE. Paired *t*-tests were used to compare MAP, HR and BRS changes before and after colorectal distension. The MAP, HR and BRS changes in response to losartan and Ang II injections at different time points were performed using repeated one-way ANOVA, with post hoc Student-Newman-Keuls test. Difference was defined as significant at *p*<0.05.

## Results

### Effect of colorectal distension on BP and HR in SCI rats

**Fig 1:**
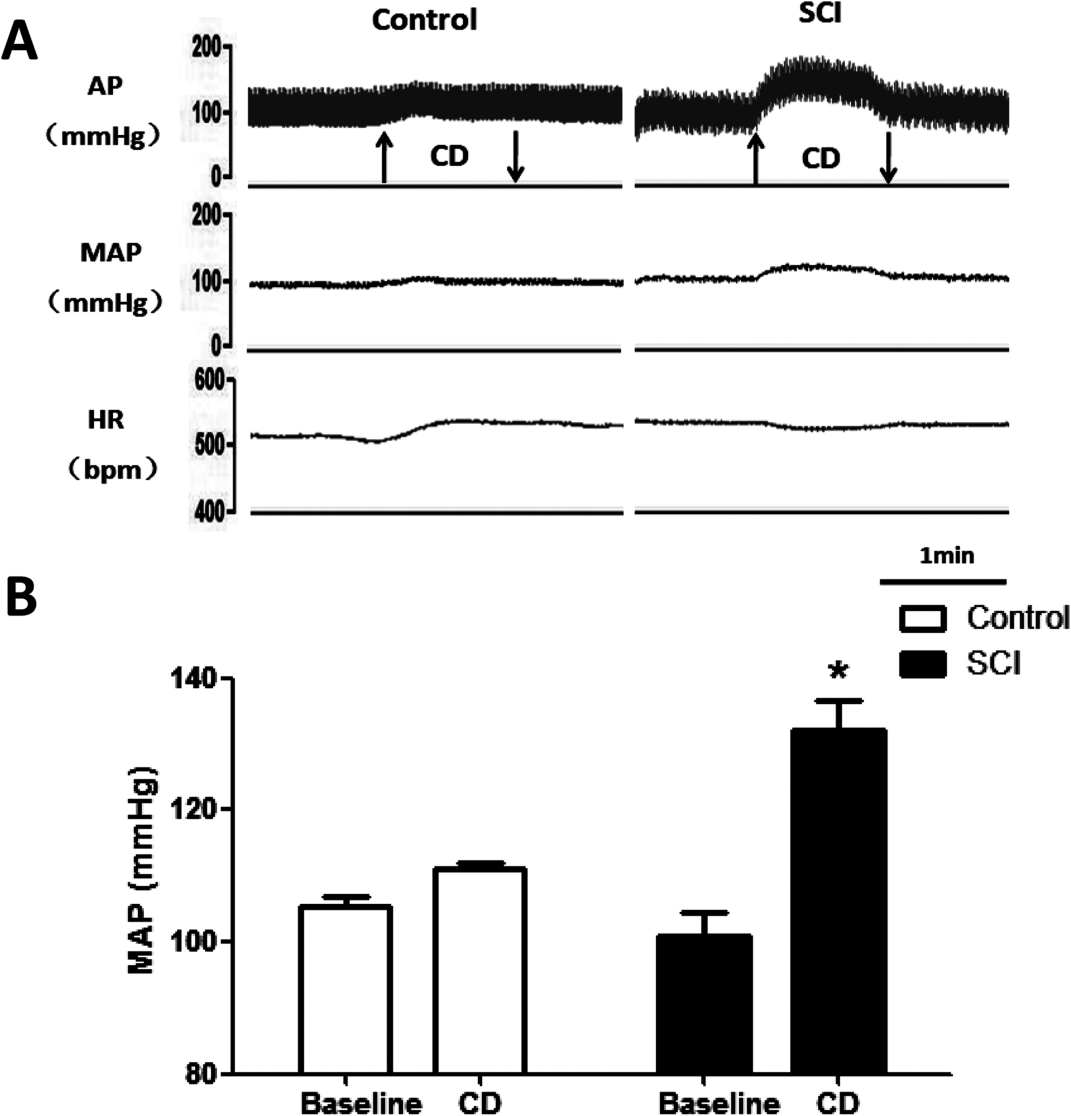
The changes in MAP and HR evoked by autonomic dysreflexia with colorectal distension (CD). (A) Original tracings showing the MAP and HR response to CD in control and SCI rats. ↑, begin of CD; ↓, end of CD;(B) bar graph showing the changes of MAP in baseline and CD. n = 5/group. * p<0.05 compared with baseline.

No significant difference was found in baseline mean arterial pressure (MAP) and HR between the SCI and the control groups(n = 5)six weeks after SCI or sham procedure. In SCI rats, MAP was increased by 31 ± 6 mmHg after colorectal distension, which indicated that AD was successfully induced. While in the control group, the MAP was only increased by 5 ± 4 mmHg after colorectal distension, which was significantly lower than that of the SCI group (*p*<0.05). Similarly, the decrease in HR (19 ± 5 vs 4 ± 3 bpm) by colorectal distension was significantly (*p*<0.05) higher in the SCI group than the control group (Fig 1 and Table 1). After these tests, the SCI rats which were successful induced AD upon colorectal distention were selected out for the following experiments.

**Table 1:** MAP and HR responses to colorectal distension (CD) in sham and SCI group.

	MAP(mmHg)		HR(bpm)	
Group	Baseline	CD	Baseline	CD
**Sham(n = 5)**	106±3	111±2	413±8	417±9
**SCI(n = 5)**	101±8	132±10*	432±49	413±49

Data were presented as mean±SE.

*P<0.05 compared with sham.

### Effect of colorectal distension on BRS

**Fig 2:**
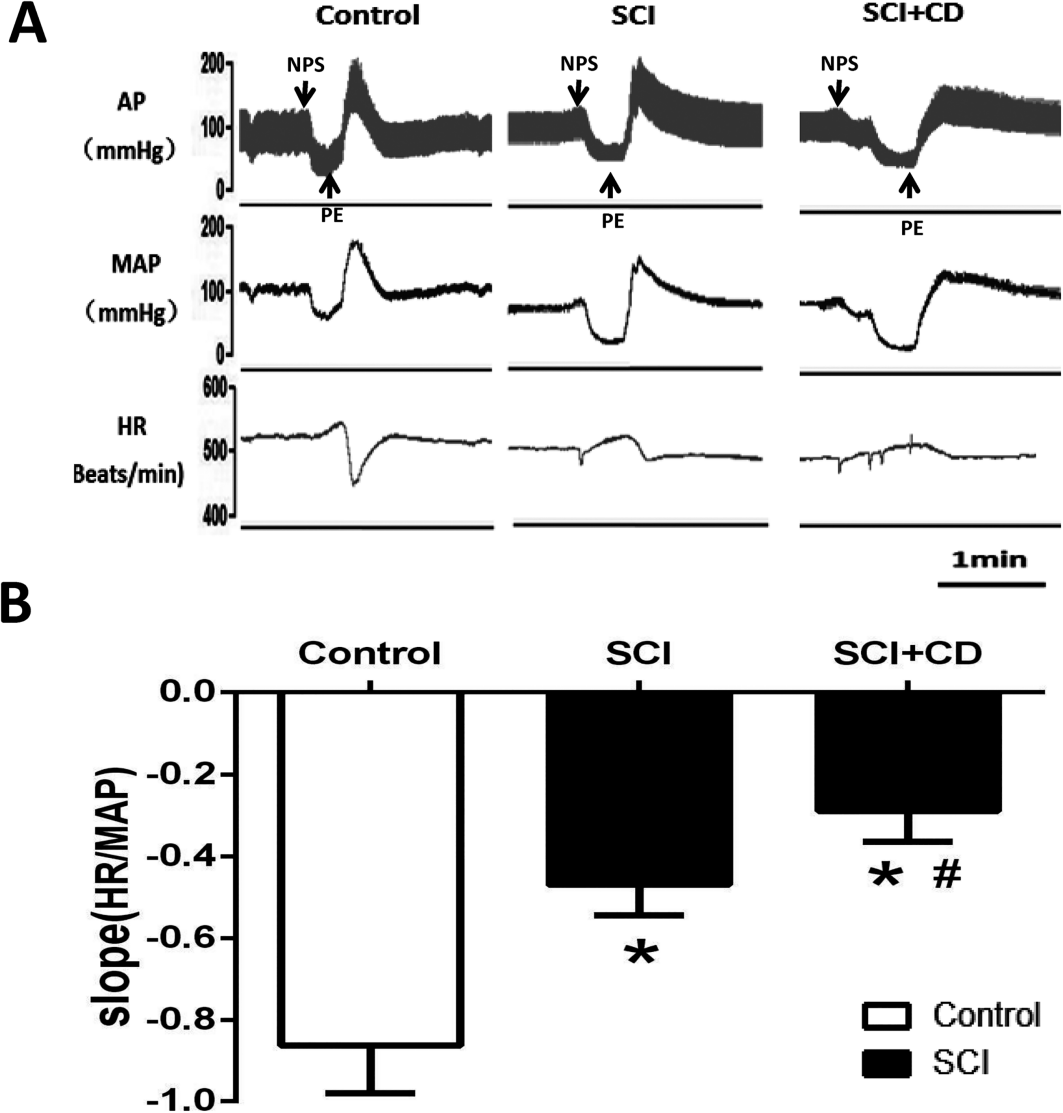
The effect of SCI and CD on baroreflex sensitivity (BRS). (A)Original tracings showing MAP and HR changes induced by intravenous nitroprusside sodium (100 μg/kg) and phenylephrine (80 μg/kg) in control and SCI rats. (B) bar graph showing the changes of BRS slop (bpm/mmHg) in control, SCI baseline and SCI+CD. n = 5/group. * p<0.05 compared with control; #p<0.05 compared with SCI.

The BRS was measured in SCI rats which were successful induced AD or sham operation rats in resting and in the response to colorectal distension. It was found that BRS in the SCI group was significantly reduced compared with the control group (-0.47 ± 0.08 vs -0.85 ± 0.14 bpm/mmHg), and it was further (*p*<0.05) blunted by colorectal distension (-0.29 ± 0.07 bpm/mmHg) (Fig 2).

### Effects of Ang II in NTS on MAP and BRS in response to colorectal distension

**Fig 3:**
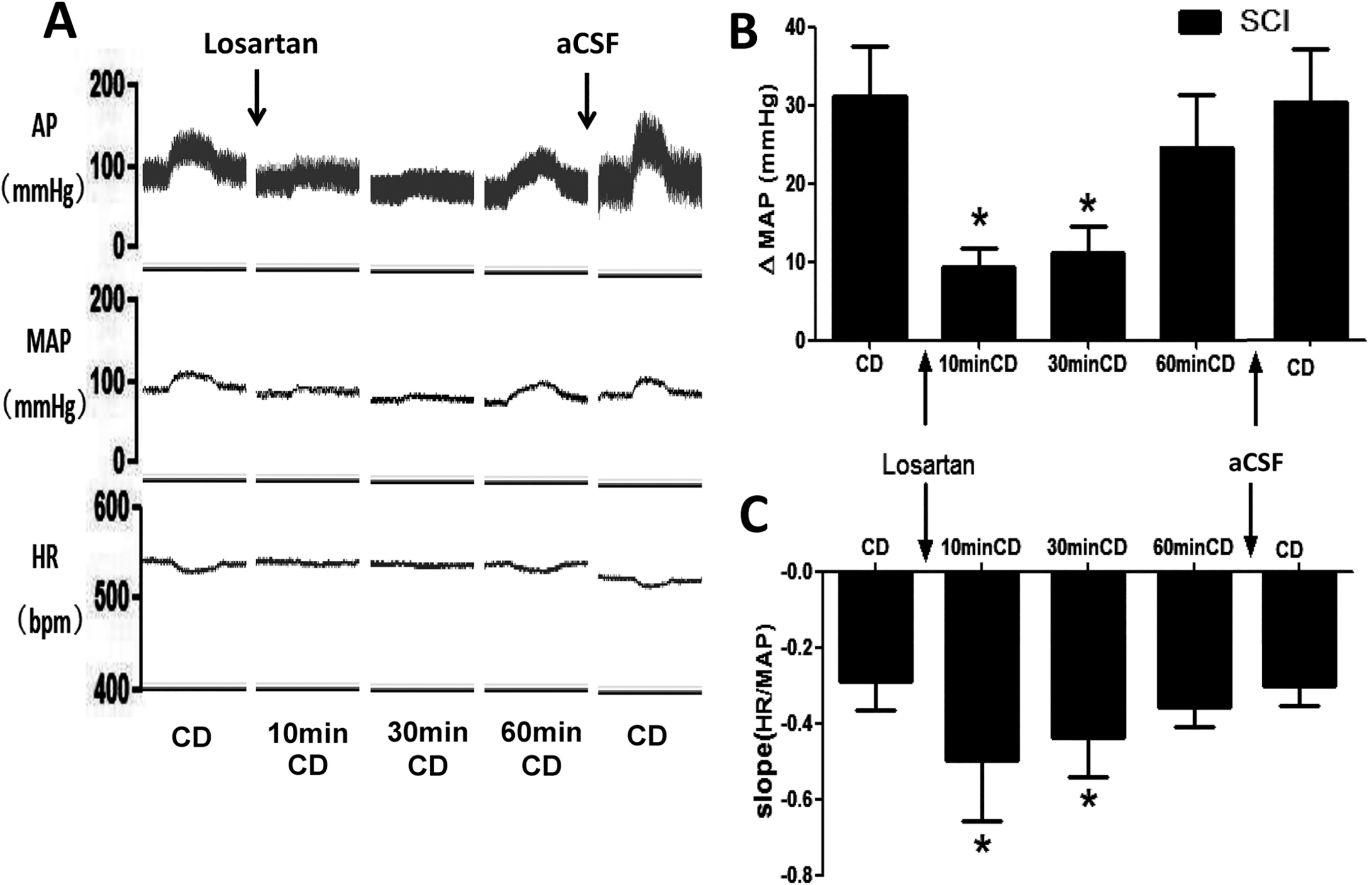
The effect of losartan microinjection on MAP and HR during CD in SCI rats. **(**A) Original tracings showing the changes in MAP and HR during CD before and after losartan microinjection into the bilateral NTS. (B-C) bar graphs showing the changes in MAP and BRS during CD before and after losartan microinjection into the bilateral NTS. n = 5/group. * p<0.05 compared with CD.

We also found that level of Ang II in the NTS was significantly increased after SCI. The changes of MAP and BRS in response to CD were measured 10, 30, and 60 min after bilateral microinjection of losartan into the NTS (n = 5). The MAP elevation induced by colorectal distension was significantly attenuated and the decreased BRS by CD was significantly improved 10 and 30min after NTS injection of losartan (Fig 3). The vehicle interference of aCSF was also explored and no difference was detected in the changes of MAP and BRS in response to CD after the injection of aCSF. In additional, we also found that level of Ang II in the NTS was significantly increased (0.39 ± 0.05 vs0.19 ± 0.02 ng/mg) in rats after SCI (n = 5).

**Fig 4:**
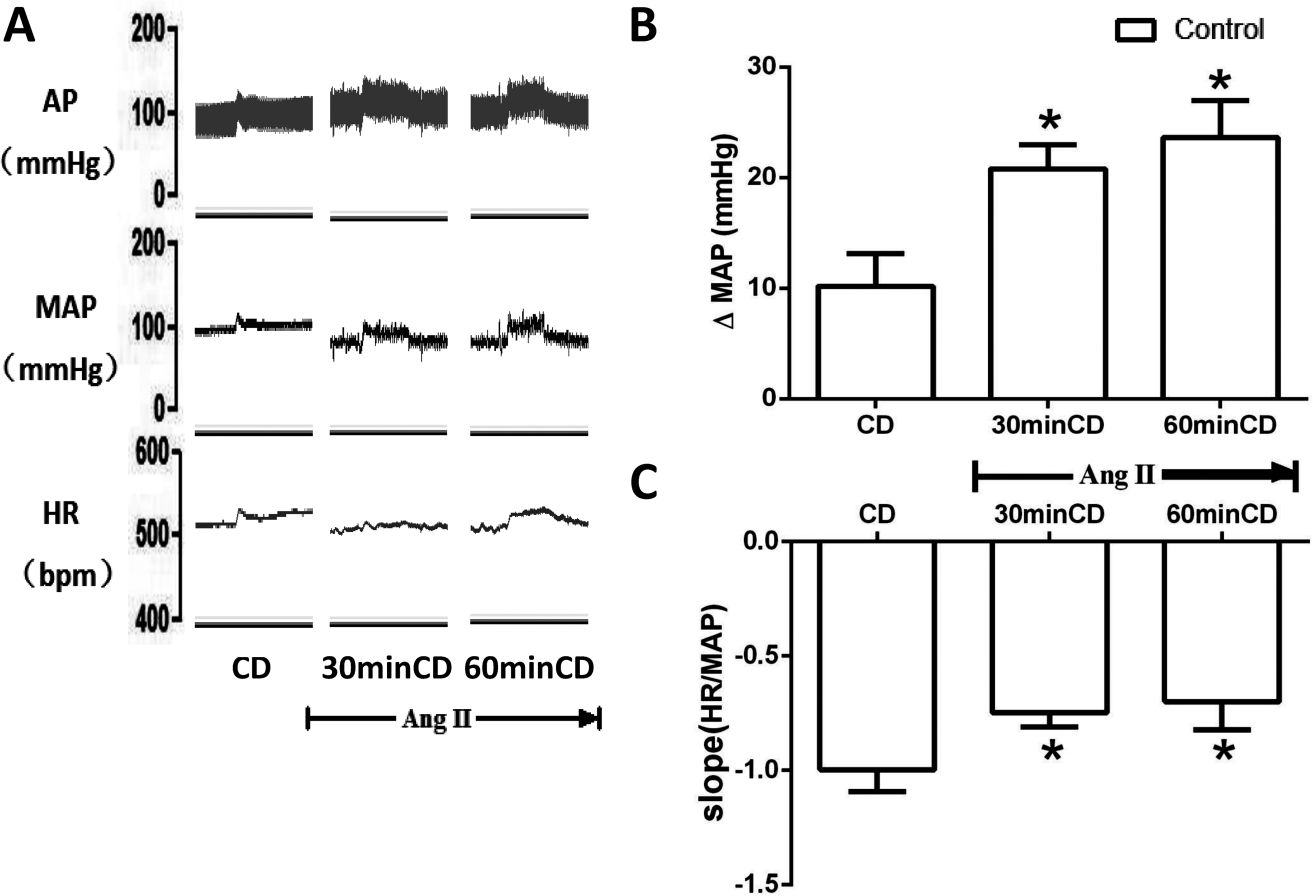
The effect of Ang II infusion on MAP and HR during CD in control rats. (A) Original tracings showing MAP and HR changes during CD before and after Ang II treatment (4th ventricle); (B-C) bar graphs showing the changes of MAP and BRS during CD before and after Ang II treatment (4th ventricle). n = 5/group. * p<0.05 compared with CD.

Control rats (n = 5) received central infusion of Ang II into the fourth ventricle to determine the effect of AT1R activation on MAP and BRS in response to colorectal distension. After Ang II treatment (30 and 60 min), the elevation of MAP and the decrease of BRS were significantly (*p*<0.05) amplified in response to colorectal distension (Fig 4).

### Effects of SAD on MAP and BRS change by colorectal distension

**Fig 5:**
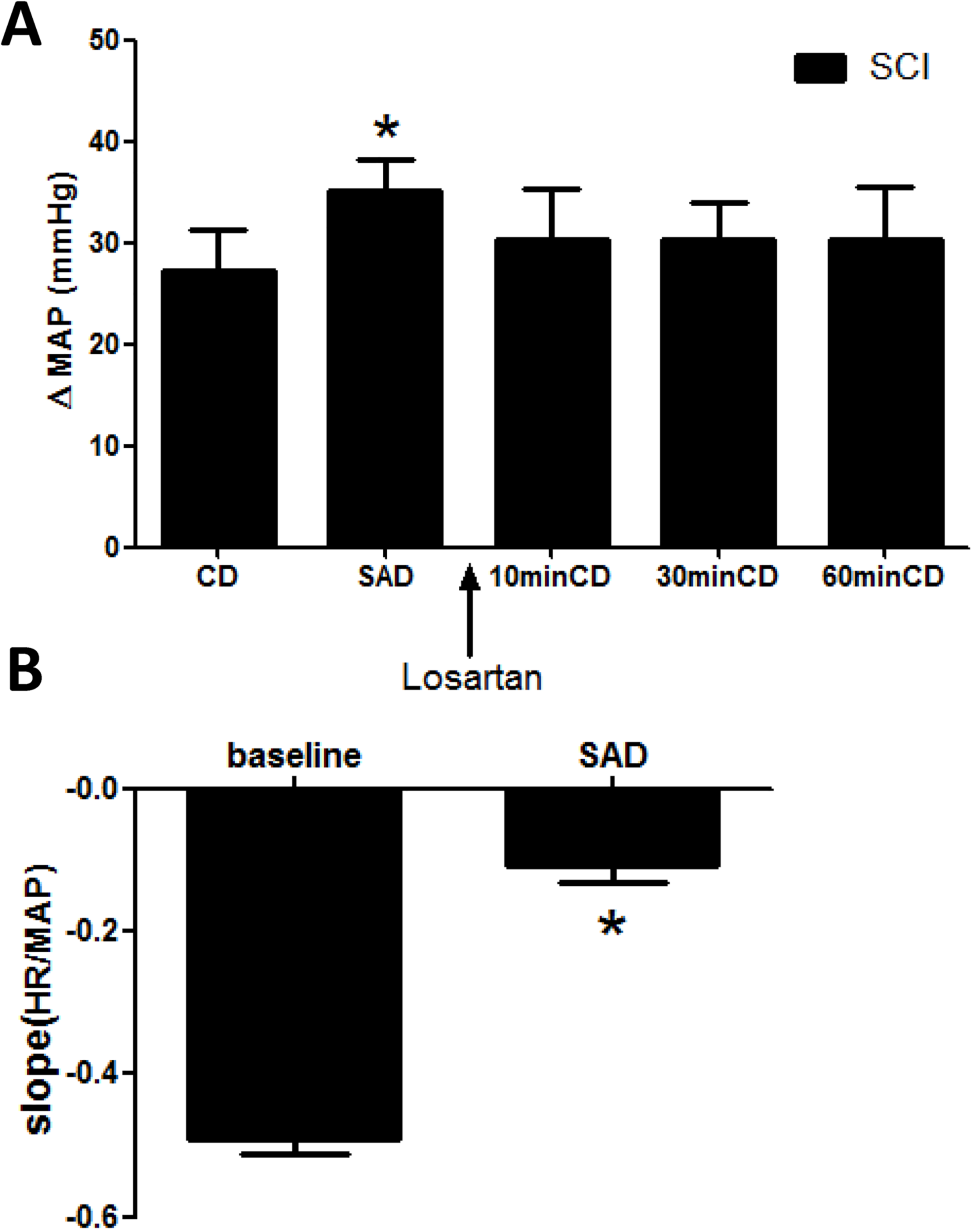
The effect of sinoaortic denervation on MAP and BRS during AD after losartan microinjection. (A) Bar graph showing that microinjection of losartan into the NTS does not attenuate MAP elevation induced by CD in SCI rats with sinoaortic denervation (SAD). *, p<0.05 compared with CD. (B) bar graph showing that BRS was decreased significantly in rats with SAD. n = 5/group. * p<0.05 compared with baseline.

SAD was performed in SCI rats to further verify the important role baroreflex plays in AD and to explore whether this effect could be blocked by microinjection of losartan into the NTS (n = 5). After SAD, the MAP increased and BRS decreased significantly (*p*<0.05), suggesting successful denervation [29]. In these SAD rats, microinjection of losartan into the NTS failed to attenuate MAP response to colorectal distension (Fig 5).

### Colocalization of AT1R and NMDAR1 receptor in the NTS neurons

As indicated in S1 Fig, the colocalization of the glutamate receptor subtype N-methyl-D-aspartic acid receptor (NMDAR) and AT1R expression was found in the same NTS neurons.

## Discussion

AD is a severe complication in the chronic stage of SCI. The underlying mechanisms still have not been well explicated. Current explanation to hypertension induced by AD is mostly focused on the peripheral system, such as that the elevated tonic activity of arterial smooth muscles and malfunction of the nerve conduction in spinal level[32–34]. It is rarely accepted that the central nervous system might play a role in AD, because seemingly sensory information cannot be transmitted from the site of irritation (for example, the colon) to the brainstem, if the spinal cord is completely transected. However, this does not necessary mean the central nervous system cannot have an impact in the onset and progression of AD. The most obvious is the control and regulation of BP baroreflex. After T4 spinal cord transection, the nociceptive transmission of spino-thalamic tract and motor transmission of corticospinal tract were cut off. Though, the anatomic structure of baroreflex and cardiac sympathetic system are retained, the autonomic nervous system also have adaptive changes with the time extension. Therefore, we aimed to test the hypothesis that the NTS might be involved in AD after SCI.

In our study, we found that microinjection of the AT1R antagonist losartan into the NTS effectively attenuated the elevation of BP response to AD triggered by colorectal distension, and improved the AD-induced blunting of BRS. Also, we have demonstrated that AT1R activation by central infusion of Ang II in control rats induceda MAP and BRS change similar to that evoked by colorectal distension after SCI. These demonstrated that Ang II system in NTS may participate in AD. Furthermore, SAD was performed to verify whether the baroreflex mechanism played a role in the above effect in SCI rats during AD.

It is known that NTS is the relay station of blood pressure regulation. When blood pressure rises, the baroreceptor transfer impulse to the NTS via sinus nerve (vagus nerve branch) and then to the dorsal vagal nucleus, which transfer impulse to vagal efferent fibers and make heart rate slow and peripheral vascular dilation. Both of these changes lead to blood pressure decrease. When blood pressure decrease, the baroreceptor transfer impulse to the NTS via the glossopharyngeal nerve and then to the sympathetic central, ventrolateral medulla (RALM). The sympathetic efferent fibers result in heart rate fast and peripheral vascular constriction, both of which bring about blood pressure elevation.

Ang II is a major vascular-nerve conduction transmitter in the NTS. Recently, studies have shown that activation of the renin-angiotensin system is an important factor in the development of neurological hypertension. After injection of Ang II into the NTS, the endothelial nitric oxide (eNOS) which is important to regulate blood pressure and heart rate is inhibited, and the release of inhibitory neurotransmitter GABA is increased. All of these shunt the neural and electrical signal of baroreceptor into NTS, inhibit the signal transduction of the vascular pressure feedback pathway, and lead to up regulation of the blood pressure set point[35,36].

The AT1R is reported to be expressed in the NTS and involved in central control of BP and baroreflex transmission[37]. Increased AngII is an important mechanism responsible for cardiovascular dysfunction in hypertension and heart failure. In this study, we also found that Ang II level in the NTS was increased in SCI rats. Our results verified the hypothesis that the activation of AT1R in the NTS contributed to the elevation of BP during AD by reduction of BRS, and blockade of AT1R by losartan significantly blunted this effect. However, the exact mechanism by which Ang II system in the NTS changed in the situation of SCI is not clear. In this work, there is a limitation that only losartan injection was used to block the AT1R in the NTS. Clearly, gene knockdown of AT1R by retroviral or lentiviral shRNA may be more specific to determine the chronic effect of AT1R in mediating the SCI-induced change in cardiovascular dysfunction. Ang II system activation can be amplified or prolonged by several factors, such as the increase of generation, decrease of Ang II resolution, up-regulation or super-sensitivity of AT1R, etc.[25,28,38–40]. It is well known that the transmitter glutamate in the NTS plays an important role in mediating resting BP and baroreflex transmission. We found that the glutamate receptor subtype NMDA and AT1R was coexpressed in the NTS neurons, suggesting a possibility that functional change of AT1R induced by SCI affects the excitatory synaptic transmission in the NTS. It is possible that SCI probably activate the renin-angiotensin system and affect the maintaining of resting MAP and its sensitivity to AD. In acute stage of SCI, the lower level of BP may result in the oxygen deficit in neurons of the brain including medulla oblongata, this may require the overactivation of Ang II system in the NTS for maintaining and keeping the resting blood pressure at the normal level. We confirmed that the expression of AT1R in the NTS was upregulated in rats with SCI. It is reported that Ang II in the NTS attenuates baroreflex functions, whereas losartan improved its sensitivity. It has been demonstrated that the BRS was severely impaired after the SCI [41]. Therefore, when AD was performed, Ang II system was activated with bolus of Ang II releasing, which resulted in further blunting of BRS.

In conclusion, the activation of Ang II system in NTS may impair blood pressure baroreflex, and contribute to the occurrence and deterioration of AD in SCI rats.

## Supporting information

S1 Fig Colocalization of AT1R and NMDAR1 receptor in the NTS neurons. Representative fluorescence staining images of colocalization (yellow color) of AT1R (green color) and NMDAR1 receptor (red color) expression in the NTS neurons.
(TIF)

S1 Data All of the data used in Figs 1–5. (XLSX)

S2 Data All of the original experimental data recorded during every experiment. (XLSX)
