# Supplementary figures and images for: Angiotensin II system in the nucleus tractus solitarii contributes to autonomic dysreflexia in rats with spinal cord injury
Source: PLoS One. 2017 Jul 24;12(7):e0181495. doi: 10.1371/journal.pone.0181495 (PMC5524360; doi:10.1371/journal.pone.0181495)

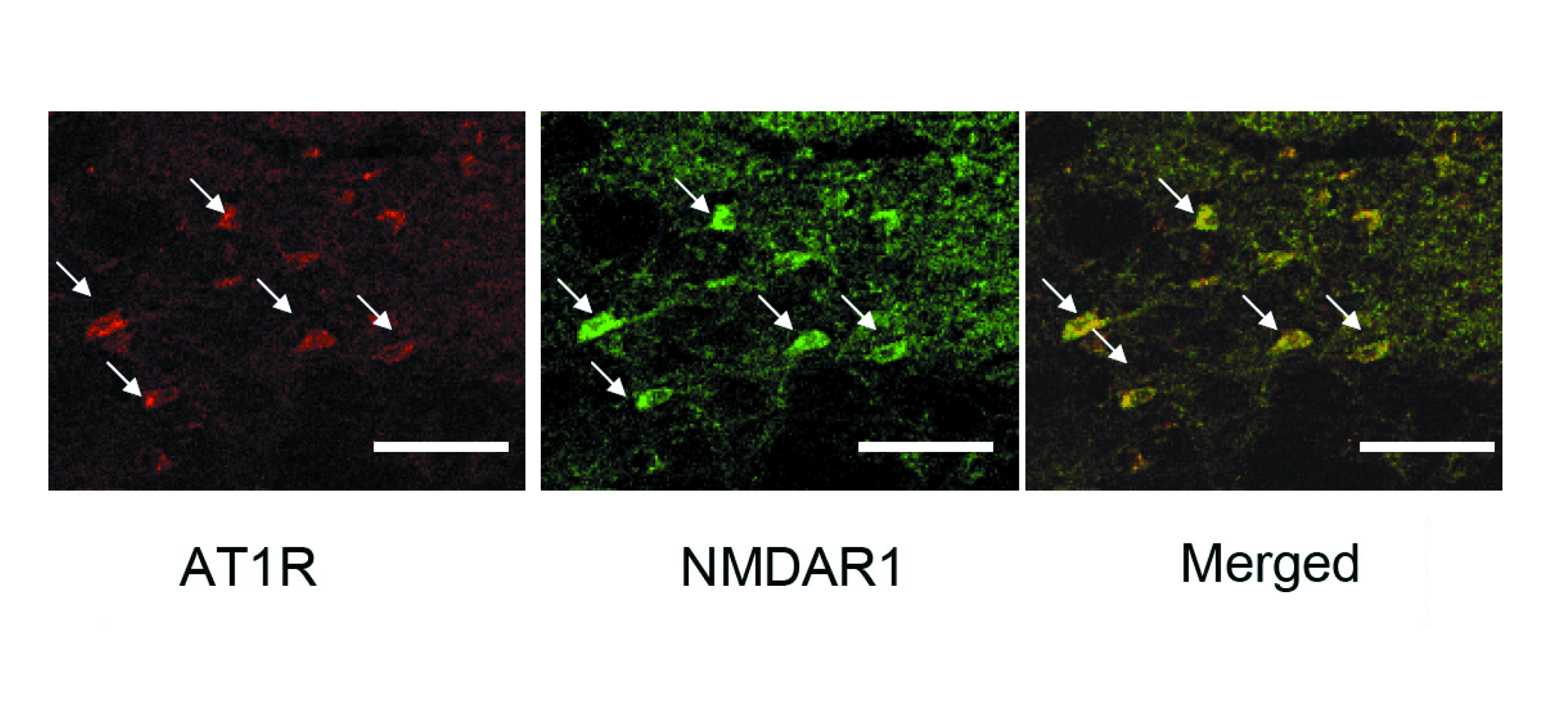

Supplement: S1 Fig — Representative fluorescence staining images of colocalization (yellow color) of AT1R (green color) and NMDAR1 receptor (red color) expression in the NTS neurons. (TIF) [file pone.0181495.s001.tif]
